# Adaptation and Psychometric Analysis of the Test of Mobile Phone Dependence—Brief Version in Italian Adolescents

**DOI:** 10.3390/ijerph18052612

**Published:** 2021-03-05

**Authors:** Rita Cerutti, Fabio Presaghi, Valentina Spensieri, Andrea Fontana, Simone Amendola

**Affiliations:** 1 Department of Dynamic and Clinical Psychology, Faculty of Medicine and Psychology, Sapienza University of Rome, 00185 Rome, Italy; valentina.spensieri@uniroma1.it (V.S.); simone.amendola@uniroma1.it (S.A.); 2 Department of Psychology of Developmental and Social Processes, Sapienza University of Rome, 00185 Rome, Italy; fabio.presaghi@uiroma1.it; 3 Department of Human Sciences, LUMSA University, 00193 Rome, Italy; andreafontanaweb@gmail.com

**Keywords:** mobile phone use, behavioral addiction, adolescents, personality functioning, sleep disturbances

## Abstract

Since the diffusion of recent models of mobile phones, anyone with an internet connection can communicate continuously and search for information. This raises some questions about the possible consequences of problematic mobile phone use (PMPU) in a complex life phase such as adolescence. Therefore, we performed a psychometric analysis of the brief version of the Test of Mobile Phone Dependence (TMD) in Italy. The sample comprised 575 Italian adolescents aged 11 to 18 years. Data were collected using the TMD-brief, the Personality Inventory for the Diagnostic and Statistical Manual of Mental Disorders (DSM-5) and the Patient-Reported Outcomes Measurement Information System (PROMIS) Sleep Disturbance Short Form. Regarding test dimensionality, the best-fit measurement model included four factors: “Abstinence”; “Abuse and interference with other activities”; “Tolerance”; and “Lack of control” (Satorra–Bentler χ^2^ (48) = 185.96, *p* < 0.01; robust root mean square error of approximation (RMSEA) = 0.079 (90% confidence interval (CI): 0.067; 0.091); robust TLI = 0.904; robust comparative fit index (CFI) = 0.930). The Italian version of the TMD-brief was found to have good reliability and psychometric properties, and a four-factorial structure. PMPU predicted significant sleep disturbances and this relationship was moderated by clinical personality traits. Findings from this study support the use of the Italian version of the TMD-brief as a screening tool to investigate PMPU in Italian adolescents.

## 1. Introduction

More than 4 billion people (60% of the world’s population) are connected to the internet [1,2]. In the last two decades, a real technological and informational revolution has taken place. The internet influences every aspect of people’s daily life, directly or indirectly, particularly with respect to communication and interaction between individuals. The International Telecommunication Union [3] estimates indicate that 70% of the world’s young people (15 to 24 years old) are online, and this proportion is significantly higher compared to that of the total population using the internet.

The possibility of continuous communication and connection goes beyond the natural concept of time and physical distance. Nevertheless, we can be in real-time communication with our interlocutor by a click of the screen and see him through a video chat even if we are physically separated by great distances. Essentially, smartphones makes a constant connection to the internet and communication with others possible. In 2019, 90% of the world’s population who accessed the internet did it through their mobile phones [2].

At the same time, this continuous connection influences concepts of private place, intimacy and privacy. Worldwide, 3.8 billion people use social media, representing 49% of the world’s population [2]. Social networks allow us to have our own profile or internet page, where we can enter and share information, images and photos, videos, contacts and friendships with others in real time (e.g., Facebook or Instagram). Moreover, this new living environment has an impact on the way in which we perceive ourselves [4].

A study conducted in different European countries (i.e., Belgium, Denmark, Ireland, Italy, Portugal, Romania and the United Kingdom), highlighted that almost half of young people aged between 9 and 16 used their own smartphone to access the internet daily [5]. Furthermore, boys and girls seemed to acquire smartphones at a similar age while the probability of children owning a smartphone increases by 58% for each year they grow older [5]. In Italy, children mainly accessed the internet from mobile phones rather than from other technological devices, with 51% of children aged 9–10 and 82% of children aged 11–12 [6]. For the most part, daily online activities involved communication with family and friends and entertainment (e.g., watching videos and visiting social networks). Compared to 2013, in 2017 there was a significant increase in using smartphones for accessing the internet among children (i.e., from 42% in 2013 to 84% in 2017) [6]. These data are in line with those observed in other countries and societies. A recent study showed that among a sample of Japanese adolescents, 91% of boys and 96% of girls had their own smartphone [7]. Furthermore, adolescents spent a considerable amount of daily time using their smartphones. The findings of the study previously cited indicated that approximately 65–70% of adolescents spent between 1 and 4 h per day using their smartphones while nearly 30% spent 4 or more hours a day [7]. Adolescents were mainly involved in the use of social networking sites and online chat but also in viewed videos, conducted internet searches and played games. These results are in line with those of a recent study conducted among 1824 Korean adolescents showing an average smartphone use of approximately 5 h per day [8]. In particular, adolescents spent half an hour per day using social networking sites and three hours for gaming and internet (except for learning) activities. The use of smartphones increases with age both in western and eastern countries [5,9]. Specifically, smartphone use for social networking sites and entertainment increase with age while its use for gaming and learning activities does not change as shown by a recent study [9]. Given the above findings, communication through mobile phones has become a fact of life for young people.

Although mobile phones are an extraordinary tool that facilitate communication, social exchanges and personal activities, an excessive, uncontrolled and inappropriate use can give rise to problems in interactions with parents and other areas of daily life functioning [10].

Problematic Mobile Phone Use and Psychophysical Well-Being

Problematic Mobile Phone Use (PMPU) is generally conceptualized as a behavioral addiction including the core components of addictive behaviors, such as cognitive salience, loss of control, mood modification, tolerance, withdrawal, conflict and relapse [11,12]. According to Chóliz’s [13] definition of dependence, PMPU represents a condition characterized by: (a) excessive use, both in terms of high economic cost and the number of calls and messages; (b) interpersonal problems associated with excessive use; (c) interference with academic or occupational activities; (d) tolerance, i.e., a gradual increase in the amount of use needed to obtain the same level of satisfaction, as well as the need to substitute operative devices with new models that appear on the market; (e) abstinence symptoms, i.e., an urgent need to use a mobile phone after some time has elapsed since its last use, as well as emotional alterations when its use is impeded or made difficult; and (f) lack of control, i.e., inability to stop the addictive behavior.

Previous studies have revealed a wide variability in prevalence rates, with percentages ranging from 2.4% to 31.3% [14,15,16,17,18,19,20]. Initial studies that investigated the potentially dangerous behaviors related to the use of mobile phones were devoted to explore its negative influence on driving control [21,22]. Research has shown that PMPU was positively associated with sedentary behavior [23], worse physical health and quality or sleep duration [24,25,26], worse attention span and school performance [27] and finally, worse quality of life linked to school and family environments [28]. Furthermore, there is evidence of a link between PMPU and dysfunctional behaviors and psychopathological symptoms such as fear of missing out (FoMO) [29], hyperactivity, poor prosocial behavior [28], low self-esteem, stress, anxiety and depression [30] and suicidal ideation and suicide attempts [19]. Recently, the Italian Pediatric Society has focused attention on the negative influences of PMPU and other technological instruments on the psychophysical development of adolescents, providing advice for parents and clinicians [31].

Research has shown the potential effects of mobile phone use on sleep quality and disturbances. Sleep problems were related to both smartphone use [32] and PMPU [33,34]. The use of smartphones, especially during bedtime, is negatively associated with different sleep characteristics among children and adolescents, such as inadequate sleep quantity, poorer quality and high daytime sleepiness [32]. These findings raise concerns as sleep plays an important role in maintaining good psychophysical health. Despite the fact that some sleep characteristics go through a period of change in adolescence, previous studies have suggested that sleep problems can have an impact on several aspects of daily functioning such as difficulties in regulating negative emotions and externalizing problems [35,36,37].

At the same time, the relationship between PMPU and sleep quality and disturbances may vary depending upon specific characteristics [38]. In other words, certain adolescents may be more susceptible to the effects of PMPU. Pathological personality traits are related to impairment in both the interpersonal and personal context as well as with addictive behaviors and problematic use of technology, including PMPU [39]. Few studies have examined the role of personality traits as moderators of the associations between the problematic use of technology and daily functioning among adolescents [40] while they are lacking for PMPU. Therefore, we examined whether the direct effect of PMPU on sleep quality and disturbances was moderated by personality traits as conceptualized by the Alternative Model of Personality Disorder provided in Section 3 of the Diagnostic and Statistical Manual of Mental Disorders (DSM-5) [41]. According to the Alternative Model, five broad domains of maladaptive personality traits reflect phenotypic variability in personality dysfunction. These five major domains include: Negative Affectivity (frequent and intense experiences of a wide range of negative emotions); Detachment (avoidance of socioemotional experience, including both withdrawal from interpersonal interactions and restricted affective experience and expression); Antagonism (behaviors that put one at odds with other people, including ideas of self-importance and callousness towards and/or unawareness of others); Disinhibition (orientation towards immediate gratification and impulsivity with no regard for past learning or future consequences); and Psychoticism (exhibiting culturally incongruent, odd, eccentric, or unusual behaviors and cognitions, both in content and process). The present study had an exploratory character considering that no previous study examined the moderator role of personality traits of the link between PMPU and sleep disturbances.

In light of the above, the present study aimed to adapt and validate a multicultural measure assessing PMPU in an Italian population, the Brief Multicultural Version of the Test of Mobile Phone Dependence (TMD-brief) questionnaire [12], as well as to analyze its psychometric properties. Choliz et al. [12] have identified a factorial structure that represents the main components of addiction: Abstinence, Abuse and interference with other activities, Tolerance and Lack of control. This factorial structure was replicated for different samples included in their study and the TMD-brief was sufficiently sensitive to detect differences among several populations with respect to PMPU. To the best of our knowledge, this is the first study to examine this topic using multiculturally validated instruments for PMPU in a population of Italian adolescents. In particular, the specific goals were: (a) to adapt a version of the TMD-brief using a translation-back-translation method in a sample of Italian participants aged 11 to 18 years old; (b) to analyze its dimensional structure using confirmatory factor analysis; and (c) to investigate the relationship between symptoms of PMPU and daily functioning, namely, personality functioning and sleep disturbances.

## 2. Materials and Methods

### 2.1. Participants and Procedure

A sample of 575 adolescents (294 males and 277 females) aged 11 to 18 years with an average age of approximately 14.1 (standard deviation (SD) = 1.86; males mean age = 14.3, SD = 1.89; female mean age = 13.9, SD = 1.81) took part in the study. Due to missing responses, 47 participants (approximately 8%) were excluded from the study. The final sample comprised 528 adolescents.

Inclusion criteria for participation in this study included the use or possession of a mobile phone or smartphone and the ability to understand the written Italian language. Exclusion criteria included a history of significant neurological illness or brain injury and the use of medications that could affect the study’s outcomes.

Participants were recruited from two secondary schools in central Italy. This was a convenience sample and the schools do not represent a random sample of the school population. The aim of the study was illustrated to the headmasters and teachers of each school, outlining that the study was designed to evaluate the psychophysical health of students. Informed consent was obtained from both participants who voluntarily participated in the study and their parents before inclusion in the study. After their consensus, questionnaires were briefly presented in the classrooms during school time. The administration lasted approximately 30 min and participants filled out the questionnaires collectively in the classroom. Confidentiality of participants was ensured by assigning each student a code with no identifiable features. Every precaution was taken to protect the privacy of research subjects and the confidentiality of their personal information. This study was approved by the Ethics Committee of the Department of Dynamic and Clinical Psychology, Sapienza University of Rome (Approval Number: 8/2017).

### 2.2. Measures

All participants completed the following questionnaires.

The TMD-brief by Choliz et al. [12] is a 12-item questionnaire assessing four latent constructs related to Mobile Phone Dependence: Abstinence (“If my mobile phone were broken for an extended period of time and took a long time to fix, I would feel very bad”; “If I don’t have my mobile phone, I feel bad”, “I don’t think I could stand spending a week without a mobile phone”); Abuse and interference with other activities (“I spend more time than I would like to talking on my mobile phone, sending SMSs, or using WhatsApp”, “I have gone to bed later or slept less because I was using my mobile phone”, “I use my mobile phone (calls, SMSs, WhatsApp...) in situations where, even though not dangerous, it is not appropriate to do so (eating, while other people are talking to me, etc.)”); Tolerance (“I need to use my mobile phone more and more often”, “When I have my mobile phone with me, I can’t stop using it”, “Since I got my mobile phone, I have increased the number of SMSs I send”); and Lack of Control (“As soon as I get up in the morning, the first thing I do is see who has called me on my mobile phone or if someone has sent me an SMS”, “When I feel lonely, I use my mobile phone (calls, SMSs, WhatsApp...)”, “I would grab my mobile phone and send a message or make a call right now”). Following the forward-back-translation procedure, all items were translated into Italian by a psychologist mother-tongue expert. The final version of the TMD-brief was then back-translated from Italian to English by an English mother-tongue expert with a degree in Psychology. A comparison of the original and back-translated version showed that no further modifications were needed. The Italian version of the TDM-brief (TDM-brief-Ita) is presented in Appendix A. Before running a confirmatory factor analysis (CFA), we conduct a preliminary exploratory factor analysis (principal components) by extracting four (oblimin) rotated factors. Results showed that the first 4 components explained approximately 72% of the total observed variance. On the first extracted component (29% explained variance), all Abstinence items saturated highly (>0.40). Similarly, on the second extracted component (20% explained variance), all Abuse items saturated highly. However, items expected to load on the third (Tolerance) and fourth (Lack of Control) components reported no clear pattern. To this aim, we decided to run a Confirmatory Factor Analysis in order to consider alternative factor structures.

The Personality Inventory for DSM-5 Brief Form (PID-5-BF) [41] investigates Negative Affect, Detachment, Antagonism, Disinhibition and Psychoticism. The PID-5-BF includes 25 items, five for each domain, with four response alternatives distributed on a four-point Likert scale, from 0 (very false/often false) to 3 (very true/often true). The Italian validation study showed good psychometric properties [42]. Average scores for each domain are considered in the present study. Reliability estimates in the present sample for the personality inventory domains were: Negative Affect Cronbach alpha = 0.68, McDonald’s omega = 0.74; Detachment Cronbach alpha = 0.62, McDonald’s omega = 0.66; Antagonism Cronbach alpha = 0.65, McDonald’s omega = 0.75; Disinhibition Cronbach alpha = 0.67, McDonald’s omega = 0.74; and Psychoticism Cronbach alpha = 0.70, McDonald’s omega = 0.76.

The Patient-Reported Outcomes Measurement Information System (PROMIS) Sleep Disturbance Short Form (PROMIS-SD) [43] is an 8-item self-report questionnaire that investigates sleep quality, sleep depth, restoration associated with sleep, difficulty falling asleep and trouble staying asleep, in the last week [44]. Participants respond on a five-point Likert scale and the total score can range from 8 to 40. Higher scores indicate more disturbed sleep. Reliability of the PROMIS-SD in the present sample was good (Cronbach alpha = 0.84; McDonald’s omega = 0.88).

### 2.3. Analysis Strategy

First, we ran a CFA with Robust estimator to investigate the latent factor structure against alternative models. In particular, fit statistics (χ^2^, comparative fit index (CFI), Tucker-Lewis Index (TLI), root mean square error of approximation (RMSEA)) for the four-factor model were compared to those of a one-factor model. Hu and Bentler [45] suggest that values of RMSEA and standardized mean square residual (SRMR) ≤0.08 (better if ≤0.05) and CFI and non-normed fit index (NNFI) values above ≥0.95 are indicative of good fit. The threshold for good construct reliability for Cronbach alpha and McDonald’s omega was set at 0.70 or above.

Second, we investigated the invariance of factor structure across gender using a multiple-group structural equation model (MG-SEM). A series of tests comparing fit statistics of hierarchical models was considered in order to investigate: configural invariance (same 4-factor model fitted on the two sub-samples with factor loadings and error variances free to vary across samples) and metric invariance (the configural model plus invariant factor loadings across the two samples and error variances free to vary).

Third, we investigated the relationships of the four TDM factors with clinical personality factors (Negative Affect, Detachment, Antagonism, Disinhibition and Psychoticism) and Sleep Disorder. In particular, we were interested in verifying whether the PID factors represented risk or protection factors in the relationship between dependence on mobile phone and sleep disturbance. In this view, we tested several moderation models considering each of the PID factors as a moderator, one at a time.

## 3. Results

Robust fit statistics for the four-factor model [12] are indicative of satisfactory fit: Satorra–Bentler χ^2^ (48) = 185.96, *p* < 0.01; robust RMSEA = 0.079 (90% confidence interval (CI): 0.067; 0.091); robust TLI = 0.904; robust CFI = 0.930. On the contrary, robust fit statistics for a one-factor model were definitely worse: Satorra–Bentler χ^2^ (54) = 275.54, *p* < 0.01; robust RMSEA = 0.098 (90% C.I.: 0.087; 0.110); robust TLI = 0.868; robust CFI = 0.892. The robust Satorra–Bentler χ^2^ difference between the two models was also significant (Δ robust Satorra–Bentler χ^2^ (8) = 88.90, *p* < 0.001), meaning that the fit of the four-factor model was significantly better than that of the one-factor model. Table 1 shows the standardized factor loadings, latent correlations and reliabilities for each of the four TDM-brief-Ita factors. As expected, all latent correlations were positive and high showing a high degree of overlap among TDM constructs.

### 3.1. Factorial Invariance of the TDM-Brief Four-Factor Structure across Gender

In order to investigate the hypothesis that the same four-factor structure is invariant across gender sub-groups, we ran MG-SEM with hierarchical invariance tests. As can be seen from the comparison of robust fit statistics for invariance tests shown in Table 2, the fit of the four-factor TDM-brief-Ita model with invariance of factor loadings across gender (metric invariance) was not significantly different from the configural model (Satorra–Bentler robust Δχ^2^ (8) = 11.88, *p* = 0.157). Also, the model with invariance of intercepts (investigating the invariance of item means across gender) showed a fit not statistically different from that of the invariance model (Satorra–Bentler robust Δχ^2^ (8) = 4.24, *p* = 0.835). Finally, the test for invariance of latent means across gender (that corresponds to a multivariate analysis of variance (MANOVA) on the latent means across gender) was also not significant (Satorra–Bentler robust Δχ^2^ (4) = 7.47, *p* = 0.113) meaning that each of the four factors have the same latent mean across gender. Thus, no differences emerged between the factor structure across gender.

### 3.2. Relationship between TDM Factors and Clinical Personality Factors and Sleep Disorder

Table 3 presents the descriptive statistics for the four TDM-brief-Ita factors, personality factors and sleep disturbance for the total sample and as a function of gender.

Comparing personality factors and sleep disturbance means across gender, we found significant differences for Negative Affect (*F*(1, 526) = 15.23, *p* < 0.001, *η^2^* = 0.028), Antagonism (*F*(1, 526) = 4.52, *p* = 0.034, *η^2^* = 0.009) and for Sleep Disorder (*F*(1, 526) = 4.73, *p* = 0.030, *η^2^* = 0.009). In contrast, the difference between males and females was not statistically significant for Detachment (*F*(1, 526) = 0.81, *p* = 0.369, *η^2^* = 0.002), Disinhibition (*F*(1, 526) = 0.52, *p* = 0.469, *η^2^* = 0.001) and Psychoticism (*F*(1, 526) = 1.78, *p* = 0.183, *η^2^* = 0.003). With regards to the relationships among the TDM factors and clinical personality factors and Sleep Disorder, we computed Spearman correlations given that all four TDM factors showed significant deviations from normality (Shapiro–Wilk test: Abstinence W = 0.91, *p* < 0.01; Abuse W = 0.96, *p* < 0.01; Tolerance W = 0.95, *p* < 0.01; Lack of Control W = 0.96).

Table 4 outlines the Spearman correlations, corrected for family-wise error rate, among TDM-brief-Ita factors, personality factors and sleep disturbance. All four TDM factors correlated positively and significantly with personality factors as well as with sleep disturbance.

### 3.3. Moderation Effect of the Personality Inventory for Diagnostic and Statistical Manual of Mental Disorders (DSM-5) Brief Form (PID-5-BF) Factors on the Relationship between TDM-Brief Factors and Sleep Disturbance

To investigate whether PID factors play a moderator role in the relationship between TDM total score and sleep disturbance, we performed a moderation model considering each PID factor one at a time. Table 5 shows the results for the five moderation models. Only two of the five moderation models reported a significant interaction effect between TDM total score and a specific PID factor. In particular, we found that Antagonism significantly increased sleep disturbance (b = 1.214, s.e. = 0.268, *p* < 0.01) that significantly moderated the effect of TDM total score on sleep disturbance (b = −0.579, s.e. = 0.243, *p* = 0.017). Simple slopes analysis showed that for low levels of Antagonism (16° percentile), the effect of TDM total score on sleep disturbance was positive and significant (b = 1.797, s.e. = 0.376, *p* < 0.001) while for high levels of Antagonism, the relationship was still positive but not significant (b = 0.651, s.e. = 0.351, *p* = 0.064). Therefore, it seems that low levels of Antagonism may constitute a risk factor and increase the effect of TDM score (i.e., levels of PMPU) on sleep disturbance. Likewise, we found that Psychoticism significantly increased sleep disturbance (b = 1.096, s.e. = 0.275, *p* < 0.01) and significantly moderated the effect between TDM total score and sleep disturbance (b = 0.503, s.e. = 0.246, *p* = 0.041). However, in this case, simple slopes analysis indicated that the effect of the moderator was in the opposite direction. In particular, for low values of Psychoticism (16° percentile), the effect on sleep disturbance was positive but not significant (b = 0.430, s.e. = 0.394, *p* = 0.275), while for high values of Psychoticism (84° percentile), the effect was positive and significant (b = 1.520, s.e. = 0.368, *p* < 0.001). Thus, in this case, high levels of Psychoticism increased the effect of the TDM score (i.e., PMPU levels) on sleep disturbance. Moreover, even if the effect of Negative Affect on sleep disturbance was positive and significant (b = 1.31, s.e. = 0.274, *p* < 0.01) as well as the direct effect of TDM scores on sleep disturbance (b = 0.923, s.e. = 0.274, *p* < 0.01), higher or lower levels of Negative Affect did not significantly increase or decrease the effect of TDM score on sleep disturbance (b = 0.386, s.e. = 0.253, *p* = 0.126). Thus, Negative Affect was not a moderator of the effect of TDM score on sleep disturbance. At the same time, we found that Detachment significantly increased sleep disturbance (b = 1.214, s.e. = 0.268, *p* < 0.01) but did not moderate the effect of TDM score on sleep disturbances (b = 0.473, s.e. = 0.269, *p* = 0.08). Finally, Disinhibition significantly increased sleep disturbance (b = 0.698, s.e. = 0.245, *p* = 0.01) but did not significantly moderate the relationship between TDM scores and sleep disturbance (b = 0.037, s.e. = 0.245, *p* = 0.88).

## 4. Discussion

The conceptualization and definition of PMPU as a behavioral addiction is a complex aspect under debate between researchers. The diagnosis of mobile phone addiction still remains controversial: there are no widely shared diagnostic criteria and moreover, it does not appear in any official diagnostic system, including the DSM-5. The use of multiple terms for referring to this condition concurs in creating further confusion for its evaluation (e.g., Problematic Mobile Phone Use, Mobile-Phone Addiction, Mobile-Phone Dependence, Problematic Smartphone Use, Smartphone Addiction). The same lack of clarity characterizes self-report questionnaires measuring PMPU, as tools developed from multiple definitions and different diagnostic criteria. Therefore, data are limited by methodological difficulties affecting both the diagnosis and the heterogeneity of diagnostic tools [12,14,46]. Considering the wide range of negative outcomes associated to the problematic use of mobile phones [19,23,24,25,26,27,28,29,30,47], validated and reliable tests for its investigation are needed.

Regarding the Italian version of the TMD-brief, our findings demonstrated good reliability and significant validity, supporting the measure as a sensitive and appropriate instrument for the assessment of the problematic use of mobile phones in adolescence. First, CFA pointed out that a four-factor model showed a better fit than the unique factor solution and was invariant across gender sub-groups. The four factors were Abstinence, Abuse and interference with other activities, Tolerance and Lack of control, confirming the four-factor structure found in the original study by Choliz et al. [12]. In the present study, these four factors positively correlated with both pathological personality traits and sleep disturbances. Therefore, the TMD-brief-Ita captures the core elements of addiction to mobile phones [13]. While several instruments exploring PMPU have been validated [48], the TMD-brief has the added value of having been originally validated in participants from seven countries representing three different continents (i.e., America, Asia, Europe) [12]. The TMD-brief and other validated scales used to examine PMPU have established criterion validity using the criteria for gambling or substance-use disorders [35]. Indeed, the factors obtained reflect behavioral addiction-related concepts such as tolerance, withdrawal, craving and negative life consequences.

Second, considering the fact that the use of mobile phones becomes problematic or addictive when it leads to negative consequences [46,49], our study found that PMPU interfered with daily activities and specifically, predicted sleep disturbances. This result is consistent with previous studies among adolescent populations [50,51,52]. A possible explanation is that mobile phone use during the evening can cause a physiological activation that makes relaxation difficult and deteriorates sleep quality [53]. Previous studies have shown the negative effect of evening exposure to computers or mobile phones’ screen light in suppressing the production of melatonin and consequently, affecting the circadian rhythm [54,55]. This result requires further attention considering the complex relationship between sleep, problematic technology use and the psychophysical health of adolescents [50,56].

Moreover, findings of the present study indicated that Antagonism and Psychoticism personality traits moderated the relationship between PMPU and sleep disturbances. Antagonism refers to behaviors that put one at odds with other people, including ideas of self-importance and callousness towards and/or unawareness of others. Results showed that low self-reported levels of Antagonism may constitute a risk factor and increase the effect of PMPU on sleep disturbances. We assume that PMPU in adolescents with low levels of antagonism (i.e., high levels of agreeableness) lead to increased prosocial online behaviors thereby disturbing sleep [57,58].

Results of moderation analyses also highlighted that high levels of Psychoticism increased the effect of PMPU on sleep disturbances. Psychoticism refers to the tendency to exhibit culturally incongruent, odd, eccentric, or unusual behaviors and cognitions, both in content and process. Considering the relationship between Psychoticism and emotion dysregulation [59], adolescents with PMPU and high levels of Psychoticism could experience an increase in sleep disturbances as a consequence of the difficulty in regulating changes in emotional experience elicited online.

Finally, Neuroticism (i.e., frequent and intense experiences of a wide range of negative emotions), Detachment (i.e., avoidance of socioemotional experience, including both withdrawal from interpersonal interactions and restricted affective experience and expression), and Disinhibition (i.e., orientation towards immediate gratification and impulsivity with no regard for past learning or future consequences) personality traits did not moderate the relationship between PMPU and sleep disturbance. These results are in line with those of a previous study reporting that PMPU significantly predicted sleep quality regardless of the level of a self-regulation trait [60].

Although different instruments for PMPU have been used [16] and adapted [61,62,63] in Italian populations of adolescents and adults, the present study on the adaptation of the TMD-brief to the Italian context has the advantage of primarily focusing attention on the adolescent population. De Pasquale et al. [61] reported the Italian validation of a 10-item questionnaire among 633 adolescents and young adults showing a one-factor structure. However, they did not examine the relationship between scores on the questionnaire and other variables. Pancani et al. [62] developed the Smartphone Impact Scale, a self-report scale to explore the cognitive, affective, social and behavioral impact of smartphone use among Italian adults. The analyses showed a seven-factor structure for the questionnaire, namely: Loss of control of smartphone use, Nomophobia, Smartphone-mediated communication, Emotion regulation through smartphone usage, Smartphone support to romantic relationships, Smartphone tasks support and Awareness of smartphone negative impact. Soraci et al. [63] tested the psychometric properties of a previous 6-item questionnaire among 205 Italian adults showing a unidimensional factor. By contrast, we specifically focused on the problematic use of smartphones among Italian adolescents. Importantly, our findings highlight the associations between PMPU as explored by the TMD-brief-Ita and measures of clinical personality traits and sleep disturbances. Therefore, we recommend the use of the TMD-brief-Ita as a screening tool to investigate the problematic use of mobile phones by Italian adolescents in both empirical research and clinical practice [31]. Findings from the present study indicate that when faced with adolescents with PMPU, clinicians should investigate both possible risk factors and negative repercussions. We showed that certain adolescents are more susceptible to the effects of PMPU on sleep disturbances. This finding may also be used to plan prevention interventions designed to improve healthy habits related to sleep and smartphone use while avoiding potential negative repercussions on school functioning.

Future research should pay attention to the differentiation of the normal and problematic use of smartphones. As pointed out by a previous study [48], a standard cut-off point establishing the PMPU has yet to be established. Furthermore, research needs to disentangle a complex issue, that is, whether the smartphone is a means to satisfy other addictions or if it is the object of the addiction itself, regardless of the applications and activities that are carried out through it. Finally, the understanding of the cause-effect relationship between PMPU and psychophysical health requires additional research efforts.

The present study has some limitations. First, the cross-sectional study design does not allow causal inferences on the relationships between the variables of interest. Second, data were collected using self-report measures, which may produce response biases due to social desirability. Third, we did not explore the specificities of PMPU (e.g., dysfunctional mobile phone use may often be related to interpersonal processes) or the degree of use of various mobile phone applications (such as gaming, gambling, social networking and shopping applications, etc.). Future studies should investigate these aspects and their differential influences on negative consequences [11]. Fourth, the sample was composed of secondary school students. Therefore, generalization of our results to other groups or populations should be made with caution.

## 5. Conclusions

The findings of the present study demonstrate that the Italian version of the TMD-brief demonstrated good psychometric properties to examine the problematic use of mobile phones in Italian adolescents. The four main factors, Abstinence, Abuse and interference with other activities, Tolerance and Lack of control, correlated with clinical personality traits and sleep disturbances. The moderation analyses indicated that PMPU predicted sleep disturbances, and that levels of Antagonism and Psychoticism influenced this relationship.

## Figures and Tables

**Table 1:** Standardized factor loadings, standard errors (s.e.), latent correlations among the four-factor Brief Multicultural Version of the Test of Mobile Phone Dependence Italian version (TDM-brief-Ita) model and corresponding reliability for each factor.

		Abstinence		Abuse		Tolerance		Lack of Control	
		λ	s.e.	λ	s.e.	λ	s.e.	λ	s.e.
1	I spend more time than I would like to talking on my mobile phone, sending SMSs, or using WhatsApp.	0.797 **	0.024						
2	I have gone to bed later or slept less because I was using my mobile phone.	0.826 **	0.020						
3	I use my mobile phone (calls, SMSs, WhatsApp, …) in situations where, even though not dangerous, it is not appropriate to do so (eating, while other people are talking to me, etc.).	0.675 **	0.030						
4	If my mobile phone were broken for an extended period of time and took a long time to fix, I would feel very bad.			0.573 **	0.037				
5	I need to use my mobile more and more often.			0.631 **	0.034				
6	If I don t have my mobile phone, I feel bad.			0.590 **	0.037				
7	When I have my mobile phone with me, I can’t stop using it.					0.821 **	0.026		
8	Since I got my mobile phone, I have increased the number of SMSs I send.					0.652 **	0.031		
9	As soon as I get up in the morning, the first thing I do is see who has called me on my mobile phone or if someone has sent me an SMS.					0.383 **	0.041		
10	I don’t think I could stand spending a week without a mobile phone.							0.528 **	0.035
11	When I feel lonely, I use my mobile phone (calls, SMSs, WhatsApp …).							0.674 **	0.027
12	I would grab my mobile phone and send a message or make a call right now.							0.593 **	0.036
	Abstinence	1.00							
	Abuse	0.743	0.042	1.00					
	Tolerance	0.891	0.032	0.853	0.041	1.00			
	Lack of Control	0.778	0.042	0.977	0.039	0.903	0.037	1.00	
	Cronbach Alpha	0.81		0.62		0.59		0.63	

** *p* < 0.01; N = 528.

**Table 2:** Robust tests for configural and metric invariance of the four-factor TDM-brief-Ita model.

	Df	AIC	BIC	χ^2^	Δχ^2^	Δ Df	Pr (Δχ^2^)
Configural Model	96	19,440	19,799	303.67			
Invariant loadings	104	19,436	19,761	315.55	11.88	8	0.157
Invariant intercepts	112	19,425	19,715	319.79	4.24	8	0.8349
Invariant latent means	116	19,424	19,697	327.26	7.47	4	0.1130

Note: Df = degree of freeedom; AIC = Akaike Information Criterion; BIC = Bayesian Information Criterion; χ2 = Chi-squared statistic; Δχ2 = difference in Chi-squared statistic between two hierarchical models; Δ Df = difference in degree of freedom between two hierarchical models; Pr (Δχ2) = *p*-value of the Δχ2.

**Table 3:** Descriptive statistics (M, SD, 25th–75th percentiles) for the four TDM factors, personality factors and sleep disturbance for the total sample (N = 528) and as function of gender.

	Male			Female			Total		
	M	SD	25th–75th	M	SD	25th–75th	M	SD	25th–75th
Abstinence	3.67	3.19	1–5	4.31	3.69	1–7	3.97	3.45	1–6
Abuse	4.07	4.67	2–6	4.67	2.86	3–7	4.36	2.88	2–6
Tolerance	3.83	2.78	2–6	4.35	2.83	2–6	4.08	2.81	2–6
Lack of Control	4.81	3.07	2–7	5.45	3.31	3–8	5.11	3.2	3–7
Negative Affect	0.86	0.62	0.4–1.2	1.08	0.67	0.6–1.6	0.97	0.65	−0.4–1.4
Detachment	0.66	0.57	0.2–1.0	0.71	0.55	0.2–1.0	0.68	0.56	0.2–1.0
Antagonism	0.55	0.52	0.2–0.8	0.45	0.48	0.0–0.6	0.50	0.50	−0.2–0.8
Disinhibition	0.87	0.64	−0.4–1.2	0.83	0.57	0.4–1.2	0.85	0.61	−0.4–1.2
Psychoticism	0.81	0.64	−0.2–1.2	0.88	0.65	0.4–1.4	0.84	0.65	−0.4–1.2
Sleep Disturbance	17.77	5.64	−13.75–21.00	18.96	6.86	−13.00–23.25	18.34	6.28	−13.00–22.00

M mean; SD standard deviation.

**Table 4:** Spearman correlations among TDM-brief-Ita factors, personality factors and Sleep Disorder.

	(1)	(2)	(3)	(4)	(5)	(6)	(7)	(8)	(9)	(10)
Abstinence (1)	1.000									
Abuse (2)	0.528 **	1.000								
Tolerance (3)	0.614 **	0.585 **	1.000							
Lack of Control (4)	0.544 **	0.611 **	0.612 **	1.000						
Negative Affect (5)	0.255 **	0.247 **	0.257 **	0.225 **	1.000					
Detachment (6)	0.187 **	0.178 **	0.270 **	0.206 **	0.547 **	1.000				
Antagonism (7)	0.200 **	0.182 **	0.217 **	0.152 *	0.362 **	0.484 **	1.000			
Disinhibition (8)	0.122	0.172 *	0.220 **	0.210 **	0.398 **	0.392 **	0.391 **	1.000		
Psychoticism (9)	0.187 **	0.194 **	0.259 **	0.213 **	0.530 **	0.566 **	0.507 **	0.516 **	1.000	
Sleep Disturbance (10)	0.169 **	0.202 **	0.147 *	0.160 *	0.217 **	0.210 **	0.153 *	0.141	0.182 **	1.000

Note. Statistical significance was adjusted for multiple tests with Bonferroni method of correction; * *p* < 0.05; ** *p* < 0.01; N = 528.

**Table 5:** Moderation effect of Personality Inventory for Diagnostic and Statistical Manual of Mental Disorders (PID) factors on the relationship between TDM-brief-Ita total score and sleep disturbance.

	Unstandardized b	s.e.	Standardized Β	*p*
Intercept	18.224	0.270	2.906	<0.001
TDM total score	0.923	0.274	0.147	0.001
Negative Affect	1.310	0.274	0.209	<0.001
TDM total score * Negative Affect	0.386	0.253	0.064	0.126
Intercept	18.228	0.268	2.907	<0.001
TDM total score	1.008	0.270	0.161	<0.001
Detachment	1.214	0.268	0.193	<0.001
TDM total score * Detachment	0.473	0.269	0.073	0.079
Intercept	18.480	0.271	2.947	<0.001
TDM total score	1.222	0.273	0.195	<0.001
Antagonism	0.841	0.285	0.134	0.003
TDM total score * Antagonism	−0.579	0.243	−0.106	0.017
Intercept	18.329	0.270	2.923	<0.001
TDM total score	1.186	0.272	0.189	<0.001
Disinhibition	0.698	0.273	0.111	0.011
TDM total score * Disinhibition	0.037	0.245	0.006	0.880
Intercept	18.197	0.270	2.902	<0.001
TDM total score	0.929	0.275	0.148	0.001
Psychoticism	1.096	0.275	0.175	<0.001
TDM total score * Psychoticism	0.503	0.246	0.087	0.041

## Data Availability

The data presented in this study are available on request from the corresponding author.

## Appendix A

**Table A1:** Italian version of TDM-brief [12].

Nr	F	Item					
1	Abs	Se il mio cellulare fosse rotto per un periodo di tempo prolungato e richiedesse molto tempo per essere riparato, mi sentirei molto male	0 Completamente in disaccordo	1	2	3	4 Completamente d’accordo
2	Abs	Se non ho il mio cellulare, mi sento male	0 Completamente in disaccordo	1	2	3	4 Completamente d’accordo
3	Abs	Non credo che potrei sopportare di trascorrere una settimana senza il cellulare	0 Completamente in disaccordo	1	2	3	4 Completamente d’accordo
4	Abu	Trascorro più tempo di quello che vorrei per parlare al cellulare, inviare SMS, o usare WhatsApp	0 Mai	1	2	3	4 Frequentemente
5	Abu	Sono andato a letto più tardi oppure ho dormito meno perché stavo usando il mio cellulare	0 Mai	1	2	3	4 Frequentemente
6	Abu	Uso il mio cellulare (chiamate, SMS, WhatsApp, …) in situazioni in cui, anche se non è pericoloso, non è opportuno farlo (mangiando, mentre altre persone mi parlano)	0 Mai	1	2	3	4 Frequentemente
7	Tol	Ho bisogno di usare il mio cellulare sempre più spesso	0 Completamente in disaccordo	1	2	3	4 Completamente d’accordo
8	Tol	Quando ho il mio cellulare con me, non riesco a smettere di usarlo	0 Completamente in disaccordo	1	2	3	4 Completamente d’accordo
9	Tol	Da quando ho il mio cellulare, ho aumentato il numero di messaggi che invio	0 Completamente in disaccordo	1	2	3	4 Completamente d’accordo
10	LoC	Non appena mi alzo al mattino, la prima cosa che faccio è vedere chi mi ha chiamato sul cellulare o se qualcuno mi ha scritto un messaggio	0 Completamente in disaccordo	1	2	3	4 Completamente d’accordo
11	LoC	Quando mi sento solo, uso il cellulare (chiamata, SMS, WhatsApp, …)	0 Completamente in disaccordo	1	2	3	4 Completamente d’accordo
12	LoC	In questo momento vorrei prendere il mio cellulare ed inviare un messaggio o fare una chiamata	0 Completamente in disaccordo	1	2	3	4 Completamente d’accordo

Note. The scale for items 4, 5, and 6 ranges from 0 (Never) to 4 (Frequently); while for all other items the response scale ranges from 0 (Completely disagree) to 4 (Completely agree); Nr = item number; F = factor; Abs = Abstinence; Abu = Abuse; Tol = Tolerance; LoC = Lack of Control.
